# Synthesis of nanocapsules blended polymeric hydrogel loaded with bupivacaine drug delivery system for local anesthetics and pain management

**DOI:** 10.1080/10717544.2021.2023702

**Published:** 2022-01-31

**Authors:** Wentao Deng, Yu Yan, Peipei Zhuang, Xiaoxu Liu, Ke Tian, Wenfang Huang, Cai Li

**Affiliations:** Department of Anesthesiology, Nanfang Hospital, Southern Medical University, Guangzhou, P. R. China

**Keywords:** Hydrogels, nanocapsules, bupivacaine, anesthetics, pain management

## Abstract

Local anesthetics are used clinically for the control of postoperative pain management. This study aimed to develop chitosan (CS) with genipin (GP) hydrogels as the hydrophilic lipid shell loaded poly(ε-caprolactone) (PC) nanocapsules as the hydrophobic polymeric core composites (CS-GP/PC) to deliver bupivacaine (BPV) for the prolongation of anesthesia and pain relief. The swelling ratio, *in vitro* degradation, and rheological properties enhancement of CS-GP/PC polymeric hydrogel. The incorporation of PC nanocapsules into CS-GP hydrogels was confirmed by SEM, FTIR, and XRD analysis. Scanning electron microscopy results demonstrated that the CS-GP hydrogels and CS-GP/PC polymeric hydrogels have a porous structure, the pore dimensions being non-uniform with diameters between 25 and 300 μm. The *in vitro* drug release profile of CS-GP/PC polymeric hydrogel has been achieved 99.2 ± 1.12% of BPV drug release in 36 h. Cellular viability was evaluated using the CCK-8 test on 3T3 fibroblast cells revealed that the obtained CS-GP/PC polymeric hydrogel with BPV exhibited no obvious cytotoxicity. The CS-GP/PC polymeric hydrogel loaded with BPV showed significant improvement in pain response compared to the control group animals for at least 7 days. When compared with BPV solution, CS-GP hydrogel and CS-GP/PC polymeric hydrogel improved the skin permeation of BPV 3-fold and 5-fold in 24 h, respectively. *In vitro* and *in vivo* results pointed out PC nanocapsules loaded CS-GP hydrogel can act as effective drug carriers, thus prolonging and enhancing the anesthetic effect of BPV. Histopathological results demonstrated the excellent biodegradability and biocompatibility of the BPV-loaded CS-GP/PC polymeric hydrogel system on 7, 14, and 21 days without neurotoxicity.

**HIGHLIGHTS**
- Preparation and characterization of CS-GP/PC polymeric hydrogel system.
- BPV-loaded CS-GP/PC exhibited prolonged *in vitro* release in PBS solution.
- Cytotoxicity of BPV-loaded CS-GP/PC polymeric hydrogel against fibroblast (3T3) cells.
- Development of CS-GP/PC a promising skin drug-delivery system for local anesthetic BPV.

## 1. Introduction

Postoperative pain management is still one of the most common problems that have largely gone unexplored (Peccora & Zhou, 2015). Clinically, local anesthetics (LA) are used to control pain after operations (including gastrointestinal surgery) or to treat other acute and chronic pain (Sandhu et al., 2021). Antipyretic analgesics (e.g. acetaminophen and celecoxib) and opioids can be used to provide relief (e.g. morphine and oxycodone). However, many drugs, particularly opioids, have serious adverse effects such as nausea, respiratory suppression, and vomiting as well as the potential to cause sensitization (Nersesyan & Slavin, 2007). However, because anesthetics have a low molecular weight, the duration of analgesia generated by a single injection is typically only very few hours, which does not meet the criteria for clinical use (Becker & Reed, 2012). The use of LA in the field of postoperative analgesia has recently received considerable attention in both scientific and clinical studies. Plain LA drugs, on the other hand, have a short duration of action. LA drugs such as lidocaine, bupivacaine (BPV), ropivacaine, and others exhibit small-molecule features such as limited action duration (1–2 h for lidocaine; 2–4 h for bupivacaine and ropivacaine) and fast relocation and encapsulation of LA agents with nanosystems (El-Boghdadly et al., 2018). As a result, the design and implementation of continuous release systems to extend the analgesic activity for days while reducing side effects are essential.

Biomaterials can be used to achieve controlled release of LA, which could lead to a safe, localized, long-acting postoperative pain treatment solution (Brigham et al., 2021). Nanocapsules, hydrogels, and nanoparticles are only a few of the biomaterial-based carriers that have been studied earlier. These polymers can prolong the release of encapsulating medications while allowing them to easily diffuse away from the injection site (Patra et al., 2018). Chitosan (CS) is a linear polysaccharide made by deacetylating chitin, a structural component present in crab exoskeletons. CS has a lot of scientific interest. Because of its features as a natural, biocompatible, bioadhesive, and biodegradable cationic polysaccharides polymer (Elgadir et al., 2015) CS hydrogels are non-cytotoxic, may be designed to gel quickly while delivering a sustained dosage, and decay slowly into biodegradable products over time, making them potential LA delivery vehicles (McLaughlin et al., 2012). Genipin (GP) has been extensively reported in the literature as a naturally present cross-linking agent for synthesizing cross-linked composites, because of its ideal properties and outstanding biodegradability, as well as the fact that it is a stable cross-linked product (Dimida et al., 2017). GP, a natural replacement to glutaraldehyde, has been proven to create non-toxic and stable polymerizing compounds that are 10,000 times less cytotoxic than glutaraldehyde (Lai, 2012). Another benefit of utilizing GP is its great selectivity, since it primarily targets main amino groups (–NH2), while the nucleophilic –OH groups (–OH and –COOH) do not react with it (Muzzarelli, 2009). The cross-linking reaction between GP and biomaterials with primary amine groups, for instance, is mild and green. However, there are few papers on GP polymerized hydrogels, which stimulates us to use GP as a crosslinking agent in the development of CS hydrogels for biological purposes.

Various ways have recently been devised for the massive manufacture of polymeric capsules, and the template method, which involves precipitated polymers on the interface of granular precursors or selectively eliminating the core of the templates, is particularly appealing (Kozlovskaya & Sukhishvili, 2006). This polymeric hydrogel capsules exhibit potential applicability in medication controlled-release systems due to the ease of manufacture, high drug loading, dual responsivity, and prolonged-release characteristics (Nan et al., 2014). Poly-ε-caprolactone (PC) is one of the most extensively used biopolymers for the manufacture of nanocapsules, because of its desired features for inclusion in semisolid drug delivery systems, such as hydrophobic nature and biocompatibility (Pohlmann et al., 2013). Polymeric nanoparticles have previously been shown to be a viable medication delivery strategy for local anesthetics. When compared to a free LA solution, articaine-loaded PC (Melo et al., 2018), PEG-PC (Grossen et al., 2017), and alginate/CS nanocapsules (Kumar et al., 2015) had higher encapsulation efficiencies and lower cytotoxicity. When compared to normal lidocaine, PC nanospheres incorporating lidocaine had lesser toxicity and better analgesic efficacy. Hydrogels of articaine-loaded PC nanocapsules were recently discovered to have enhanced *in vitro* permeability along a cellulose substrate, which might lead to improved clinical status since a strong association between *in vitro* permeation factors and *in vivo* intraoral LA effect was previously found (Muniz et al., 2018; da Silva et al., 2021). The design of PC nanocapsules suspension-loaded CS-GP hydrogels is largely conventional, consisting of the dispersion of nanosuspension in a natural or synthetic polymer matrix to form a CS-GP hydrogels network. This novel PC nanocapsules suspension-loaded CS-GP hydrogels design could provide advantages in terms of innovation and application. In addition, this work focused on the development of CS-GP/PC polymeric hydrogel without the use of a polymer matrix as a platform for drug delivery.

**Scheme 1.**
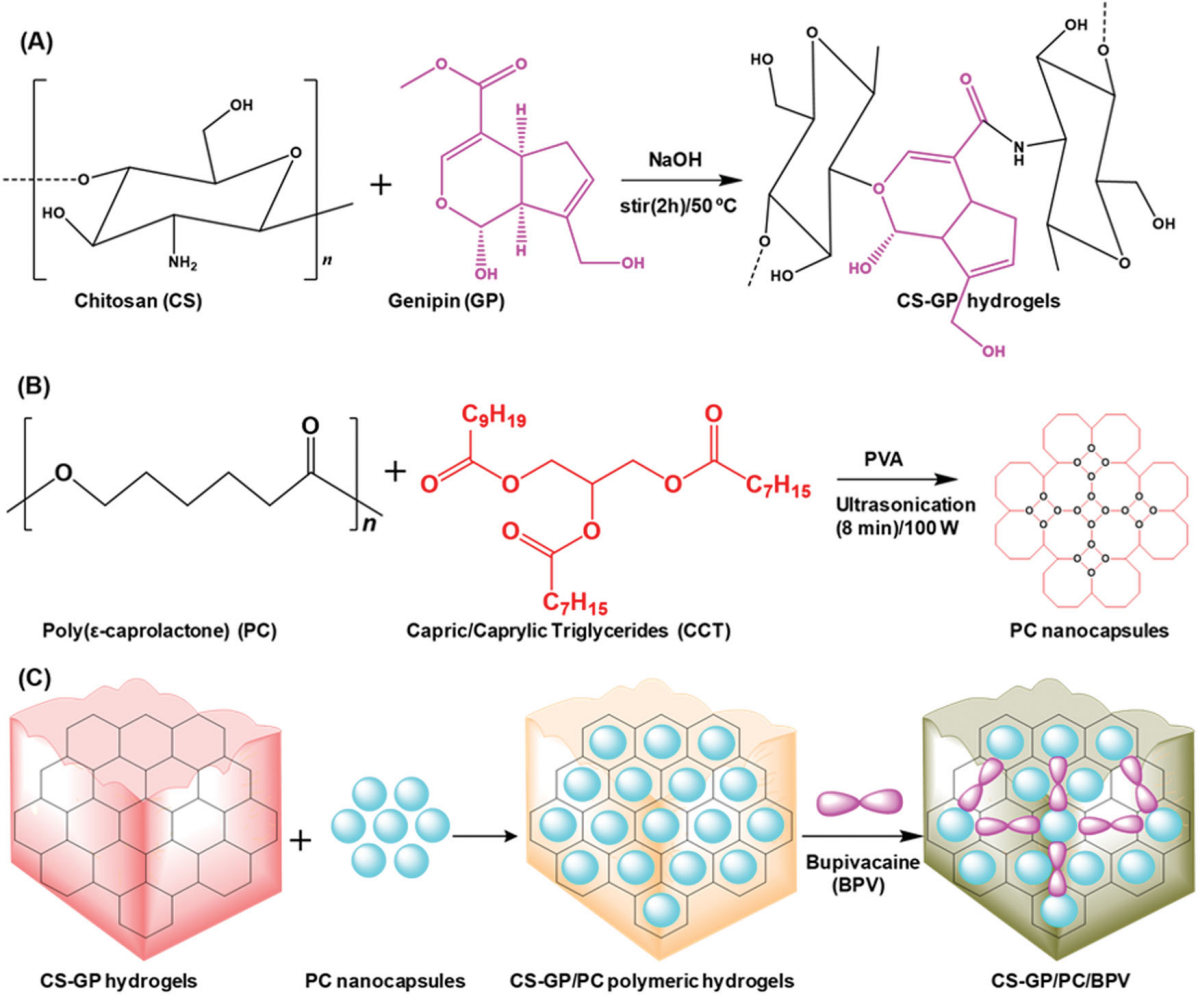
Schematic representation of (A) the preparation of chitosan (CS) and genipin (GP) crosslinked CS-GP hydrogels, (B) the preparation of PC nanocapsules, and (C) CS-GP hydrogels loaded PC nanocapsules to bupivacaine (BPV) drug delivery system for local anesthetics and pain management.

The overall objective of the present study was to determine an injectable local anesthetic-loaded drug delivery system that can provide sustained analgesic effects to minimize the number of injections and the associated side effects. In this work, chitosan with genipin (CS-GP) hydrogels loaded poly(ε-caprolactone) nanocapsules were employed to develop CS-GP/PC polymeric hydrogel to deliver with or without BPV (Scheme 1). The physicochemical and mechanical properties of CS-GP/PC polymeric hydrogel were evaluated for their cross-linking surface properties, porous morphology, *in vitro* drug release profile, gelation time, and swelling ratio. *In vitro* skin permeation and *in vivo* therapeutic effect using histopathological analysis in an animal model were further investigated.

## 2. Experimental section

### 2.1. Materials

Chitosan (CS; degree of deacetylation 75–85%), Genipin (GP), Poly(ε-caprolactone) (PCL; average Mw 14 KDa), and Bupivacaine hydrochloride (BPV) were purchased from Sigma–Aldrich. The majority of the reagents and chemicals were analytical or high-performance liquid chromatography (HPLC) grade, and they were employed without additional purification. Double distilled water was used to make aqueous solutions.

### 2.2. Synthesis of CS-GP hydrogel

The CS-GP hydrogels used in this study were synthesized by a solvent evaporation technique (Cui et al., 2014). CS was dissolved in 0.075 M glacial acetic acid at a final concentration of 2% (w/v). This mixture was titrated for several hours until it became clear. Using a 5 M NaOH solution and steady shaking, the pH of each sample was gradually raised, minimizing rapid dissolution. An appropriate volume of 0.5%, 1.0%, and 1.5% (w/v) aqueous GP solution was then added to the CS solutions to obtain a final concentration of 0.1% (w/v) of GP. Both solutions were then combined and agitated for 2 h at 50 °C to obtain a homogenous mixture solution. The air bubble-free mixtures were dropped into a shallow disk and dried in the air after being properly stirred and sonicated. The samples were submerged in NaOH solution for 2 h and then washed with deionized water until pH was neutral to remove any leftover acetic acid. Subsequently, the samples were kept in the air for 24 h before being dried under vacuum at 37 °C. Moreover, the CS-GP hydrogels suspensions were stored overnight at 4 °C to allow complete gelation. The prepared CS-GP hydrogels using various concentrations of GP (0.5%, 1.0%, and 1.5%) is named as CS-GP-1, CS-GP-2, and CS-GP-3 hydrogels.

### 2.3. Preparation of PC nanocapsules

PC nanocapsules formulations were prepared by the oil-in-water emulsion/solvent evaporation technique (Zhou et al., 2010) based on the composition reported previously (Master et al., 2010). Two solutions were mixed, one comprising 250 mg poly(ε-caprolactone) and 150 mg capric/caprylic triglycerides in 20 mL chloroform. Ultrasonication was used to create the pre-emulsion (1 min, 100 W). To make the emulsion, this pre-emulsion was mixed with 25 mL of an aqueous solution containing 75 mg of polyvinyl alcohol surfactant and sonicated for 8 min. The resultant solution was using a rotary evaporator to separate the solvents. The resultant PC nanocapsules were stored in a dryer at 4 °C before use.

### 2.4. Incorporation of PC nanocapsules into the CS-GP hydrogel

The PC nanocapsules-loaded CS-GP hydrogel was prepared according to the reported method with slight modification (Contri et al., 2014). Briefly, CS-GP hydrogels with PC nanocapsules suspension and/or water were added to the CS-GP-1, CS-GP-2, and CS-GP-3 hydrogels at a fixed concentration of 3.0%. The CS-GP hydrogels and PC nanocapsules were previously prepared for the homogenous systems (Sections 2.2 and 2.3). With the addition of lactic acid, the dispersion was acidified to a desired concentration of 1%. The CS-GP/PC polymeric hydrogel was created through manual mixing. The CS-GP/PC polymeric hydrogel was finally obtained after several centrifuging/washing cycles with water. The resultant CS-GP/PC polymeric hydrogel samples were stored in a dryer at 4 °C before use.

### 2.5. Characterizations of morphological and structural analyses

The CS-GP hydrogel, PC nanocapsules, and CS-GP/PC polymeric hydrogel and their morphology were analyzed, after complete drying of the water on a smooth glass surface, by scanning electron microscopy (SEM, JEOL JSM-5600LV) at an accelerated voltage of 10–20 kV. FT-IR spectra were performed using a Perkin-Elmer 6000 FT-IR spectrometer by using the KBr pellet technique (resolution at 4 cm^−1^, 16 scans, and wavenumber in the range of 4000–400 cm^−1^). The X-ray diffraction patterns of CS-GP hydrogel, PC nanocapsules, and CS-GP/PC polymeric hydrogel were determined using a wide-angle X-ray Simens D5000 diffractometer and Kα, Cu radiation.

### 2.6. Swelling ratio

The following method was used to study the swelling of the CS-GP hydrogel and the CS-GP/PC polymeric hydrogel. The hydrogel swelling behavior in cell-conditioned media was studied using a mixture of CS-GP hydrogel and CS-GP/PC polymeric hydrogel. The % swelling as a function of time at 37 °C was calculated using the dry mass of the CS-GP hydrogel and CS-GP/PC polymeric hydrogel. The weight of the air-dried CS-GP hydrogel and CS-GP/PC polymeric hydrogel in cell-conditioned media was observed at various time intervals (1, 3, 5, 7, 10, 14, 18, 21, and 24 h) hydrogel sample mass was recorded. The swelling ratio (SR) of the gels was calculated according to the equation: SR = (*W*_S_ − *W*_0_)/*W*_0_ × 100%. The weight of the swollen gel was *W*_S_, while the weight of the original gel was *W*_0_. Each study of swelling was done three times, with the average values given.

### 2.7. *In vitro* degradation

In the vial with a diameter of 16 mm, 1 mL of 15 wt% CS-GP/PC polymeric hydrogel solution with 20 mg of BPV was added. The mixture was then placed in a thermostat for 10 min at 37 °C to create CS-GP/PC/BPV. The weight of CS-GP/PC/BPV was then measured. The vial was then incubated in a 37 °C shaking incubator at 100 rpm with 2.0 mL of PBS without or with 2.0 mg/mL of elastase gently applied to the surface of CS-GP/PC/BPV. At each predetermined time interval (4, 8, 12, 16, 20, and 24 days), 2.0 mL of clear supernatant was taken, and the remaining CS-GP/PC polymeric hydrogel was correctly weighed before being supplemented with an equivalent volume of fresh PBS. For the examination of the degradation profile, the weight loss of CS-GP/PC/BPV was observed throughout the process.

### 2.8. Rheological properties

Using a TA Instruments AR2000 stress-controlled rheometer, the rheological characteristics of CS-GP hydrogel and CS-GP/PC polymeric hydrogel were assessed (USA). The 60 mm diameter and 2.0 mm thick gel samples were carefully removed from the Petri dish and transferred to the rheometer’s bottom plate before the rheometry testing. The temperature of the plate was set at 37 °C. The top plate was decreased to 1.5 mm in height. The evolution of storage and loss moduli (G′, G′′, respectively) as a function of time was studied throughout the gelation process at 37 °C. A frequency sweep test and a strain sweep test were carried out. The dynamic storage modulus (G′) and loss modulus (G′′) were measured at 1% shear strain amplitude at frequencies ranging from 0.1 to 100 rad/s for frequency sweep tests. The moduli were measured in strain sweep tests at 5 Hz with shear strains ranging from 0.1% to 10%.

### 2.9. Determination of encapsulation efficiency and drug loading

The drug loading was measured using an Agilent Technology (1260 Infinity) system and reversed-phase high-performance liquid chromatography (RP-HPLC). The drug loading (DL) and encapsulation efficiency (EE) of the BPV-loaded CS-GP hydrogel (CS-GP/BPV) and BPV-loaded CS-GP/PC polymeric hydrogel (CS-GP/PC/BPV) were determined using the Sephadex G50 column (GE Healthcare Bio-Sciences, Pittsburgh, PA). At a flow rate of 0.5 mL/min, the mobile phase was made up of acetonitrile and phosphate buffer solution in a 1:1 ratio. The concentration of free BPV was determined using HPLC on an Agilent series using a zorbax RP-select B column after it was isolated from the CS-GP hydrogel and BPV-loaded CS-GP/PC polymeric hydrogel. The sample solution was injected in a volume of 20 liters, with a detection wavelength of 245 nanometers. Before and after filtering, the amount of BPV-loaded CS-GP/PC polymeric hydrogel was measured. The BPV-loaded CS-GP/PC polymeric hydrogel was then dissolved in acetonitrile for an hour to break it down, and the resulting solution was filtered through a 0.2 μm filter before being injected into the HPLC column. The flow rate was 1 mL/min. The EE and DL were calculated using the following equations:

$$\mathrm{EE}\;(\%)=\frac{W_{\text{total BPV }}-W_{\text{free BPV }}}{W_{\text{total BPV }}}\times 100$$

$$\mathrm{DL}\;(\%)=\frac{W_{\text{total BPV }}-W_{\text{free BPV }}}{W_{\text{lipid }}}\times 100$$

where the *W*_total BPV_ is the total amount of BPV determined in the CS-GP/PC polymeric hydrogel system, the *W*_free_ BPV is the amount of free BPV determined in the aqueous phase after separation of the CS-GP/PC polymeric hydrogel and *W*_lipid_ is the weight of the lipid phase.

### 2.10. *In vitro* drug release

The release study of BPV drugs from the CS-GP and CS-GP/PC polymeric hydrogel was evaluated in the sink conditions, (83% PBS, 1% tween 80, and 16% methanol) at pH 7.4 and 37 °C. UV–visible spectrophotometer was used to determine the concentration of 1 mL of CS-GP/PC polymeric hydrogel solution at pre-determined time intervals (4, 8, 12, 16, 20, 24, 28, 32, and 36 h) to maintain sink conditions. After each CS-GP/PC polymeric hydrogel sampling, fresh 5 mL of PBS was added after each time interval when the aliquots had been taken to maintain the sink condition and kept it again in orbital shaking incubator up to next time interval and so on. Finally, the HPLC was used to quantify of released BPV drug molecule from the prepared CS-GP and CS-GP/PC polymeric hydrogel formulations. All of the experiments were done three times. The efficiency of BPV release was calculated as follows:

$$\mathrm{Release}\;(\%)=\frac{\text{Amount of BPV released }(\mathrm{mg})}{\text{Amount of CS}-\mathrm{GP}/\mathrm{PC}/\mathrm{BPV}\;(\mathrm{mg})}\times 100$$

### 2.11. Cell viability test

The CCK-8 assay was used to assess the *in vitro* cytotoxicity of pharmaceuticals encapsulated in CS-GP/PC polymeric hydrogel and drugs solution (CS-GP/PC/BPV) in cultures of 3T3 fibroblast cells. Cells were seeded at a density of 104 cells per well in a 48-well plate, then incubated for 24 h at 37 °C in DMEM supplemented with 10% FBS and antibiotics under 5% CO_2_. The culture medium was then removed and replaced with 100 µL of CS-GP/PC polymeric hydrogel or CS-GP/PC/BPV solution (50, 100, 150, 200, 250, and 300 μg/mL). As controls, cells that had not been treated were employed. The medium was withdrawn and the plate was rinsed with phosphate-buffered saline after the exposure duration (2 h) (pH 7.4). Each well received 100 mL of media without serum and 0.5 mg/mL of CCK-8 reagent, which was incubated for 3 h at 37 °C. Each well’s CCK-8 solution was discarded, and 100 µL of ethanol was added to dissolve the formazan crystals. Cell viability was determined using the equation;

$$\text{Cell viability }(\%)=\frac{\text{Absorbance of cells treated with samples}}{\text{Absorbance of cells treated with fresh medium}}\times 100$$

### 2.12. *In vivo* analyses

Female Sprague-Dawley (SD) pathogen-free rats (weighting 250–400 g) were purchased from the Department of Anesthesiology, Shenzhen Children’s Hospital (Shenzhen, PR China). On a 12/12 h light/dark cycle, the animals were kept in a temperature (22–24 °C) and humidity (60 ± 5%) regulated vivarium. Food and drink were freely available. All animal trials followed the Ministry of Health of the People’s Republic of China’s Animal Management Rules.

#### 2.12.1. *In vitro* skin penetration

Vertical Franz-type diffusion cells with a cross-sectional area of 6 cm^2^ and a cell volume of 7.0 mL were used *in vitro* skin permeation investigations. A peritoneal injection of Carbrital was used to anesthetize rats. To avoid damaging the stratum corneum, the fur on the abdomen area of the mice was gently shaved with an electrical shaver. The adhering fat and subcutaneous tissue were removed along with the skin from the abdomen surface. The dorsal side of the stripped skin was pointing upwards and knotted at the donor compartment. *In vitro* skin permeation tests of BPV from various formulations were conducted in a Franz glass diffusion cell at 37 °C under non-occlusive circumstances. At 37 ± 0.5 °C, the receptor compartment was filled with 7 mL of PBS (pH 7.0) containing 2% (w/w) anhydrous ethanol, and the solution was constantly agitated at 200 rpm by a magnetic stirrer. On the epidermal surface of the skin, BPV drugs loaded CS-GP hydrogel, BPV drugs loaded CS-GP/PC polymeric hydrogel, and free BPV drug (each formula contains 2% BPV) formulations were applied. At 2, 4, 6, 8, 12, 16, 20, 24, 28, 32, 36, 40, 44, and 48 h, 0.5 mL aliquots were extracted. After each sampling, the same amount of PBS solution kept at 37 °C was replaced in the receptor compartment. HPLC analysis was used to determine the samples, as mentioned in Section 2.9.

The flux of BPV drugs from each formulation was calculated. The cumulative BPV drug permeation (*Q_t_*) was calculated as follows:

$$Q_{t}=V_{r}C_{t}\sum_{i=0}^{t-1}V_{s}C_{i}$$

where *C_t_* is the BPV drug concentration of the receptor fluid at each CS-GP hydrogel and CS-GP/PC polymeric hydrogel sampling time; *C_i_* is the BPV drug concentration of the *i*th CS-GP hydrogel and CS-GP/PC polymeric hydrogel sample; *V_r_* and *V_s_* are the volumes of the receptor fluid.

The cumulative BPV drug permeability per unit of skin surface area, *Q_t_*/*S* (*S* = 0.75 cm^2^), was calculated. As illustrated in the following equation, the steady-state fluxes (JSS) were computed by linear regression interpolation of the experimental data at steady state (*T*).:

$$J_{SS}=Q_{t}/(\Delta T\;\times S)$$

#### 2.12.2. *In vivo* anesthetics pain relief evaluation

The tail-flick test was used to determine *in vivo* anesthetic pain reduction. A noxious heat stimulus was provided to the dorsal surface of the tail using a concentrated, radiant heat light source. BPV drugs loaded CS-GP hydrogel, BPV drugs loaded CS-GP/PC polymeric hydrogel, and free BPV drug (each formula includes 2% BPV) were all administered to the dorsal surface of the tail individually, as was the normal saline solution. After 5 min of local application of BPV drugs loaded CS-GP hydrogel, BPV drugs loaded CS-GP/PC polymeric hydrogel, and free BPV drug, as well as a normal saline solution control, the tail-flick test began. Each group’s pain threshold was assessed before and after treatment at predetermined time intervals (5, 10, 15, 20, 25, 30, 35, 40, 45, 50, 55, 60, 65, 70, and 75 min). The anesthetic pain reduction effect was expressed as a percentage of the maximum potential effect (%MPE). The mean of three separate measurements made at 5-min intervals was used to calculate the predrug latency (baseline tail-flick latency). To avoid tissue damage in analgesic animals, a maximum cutoff latency of 10 s was chosen. The percent MPE was computed using the following formula: %MPE = (postdrug latency - predrug latency)/(cutoff latency - predrug latency) × 100. The results are presented as the mean standard deviation of eight rats per group.

#### 2.12.3. Tissue collection and histological analysis

After 7 days, the animals were terminated by isoflurane anesthesia and intracardiac Euthanasia solution. To check for symptoms of gross pathology, the surgical site was opened and exposed. The sciatic nerve and surrounding tissues were taken from the injection site, with or without leftover BPV-loaded CS-GP hydrogel and BPV-loaded CS-GP/PC polymeric hydrogel. For 48 h, the separated tissues were fixed in 10% buffered formalin phosphate. The tissues were then cleaned in PBS before being transferred to 70% histology grade ethanol. Hematoxylin and eosin (H&E) and Masson’s trichrome were used to stain the samples, which were fixed in 10% buffered formalin, paraffin-embedded, sectioned, and stained (MST). At a magnification of 400, photomicrographs of stained BPV-loaded CS-GP hydrogel and BPV-loaded CS-GP/PC polymeric hydrogel samples on 7, 14, and 21 days were studied under a microscope. The tissue sections were taken with a Nikon DS-Fil digital camera and Nikon Elements software on an Axioskop light microscope (New York).

### 2.13. Statistical analysis

All of the tests were carried out three times. The data were presented as means ± standard deviations. In SPSS 23.0, statistical differences were assessed using paired student’s *t*-tests (SPSS Inc., Chicago, IL, USA). When the *p*-value was less than .05, differences between experimental groups were considered significant.

## 3. Results and discussion

### 3.1. Morphological analysis

**Figure 1.**
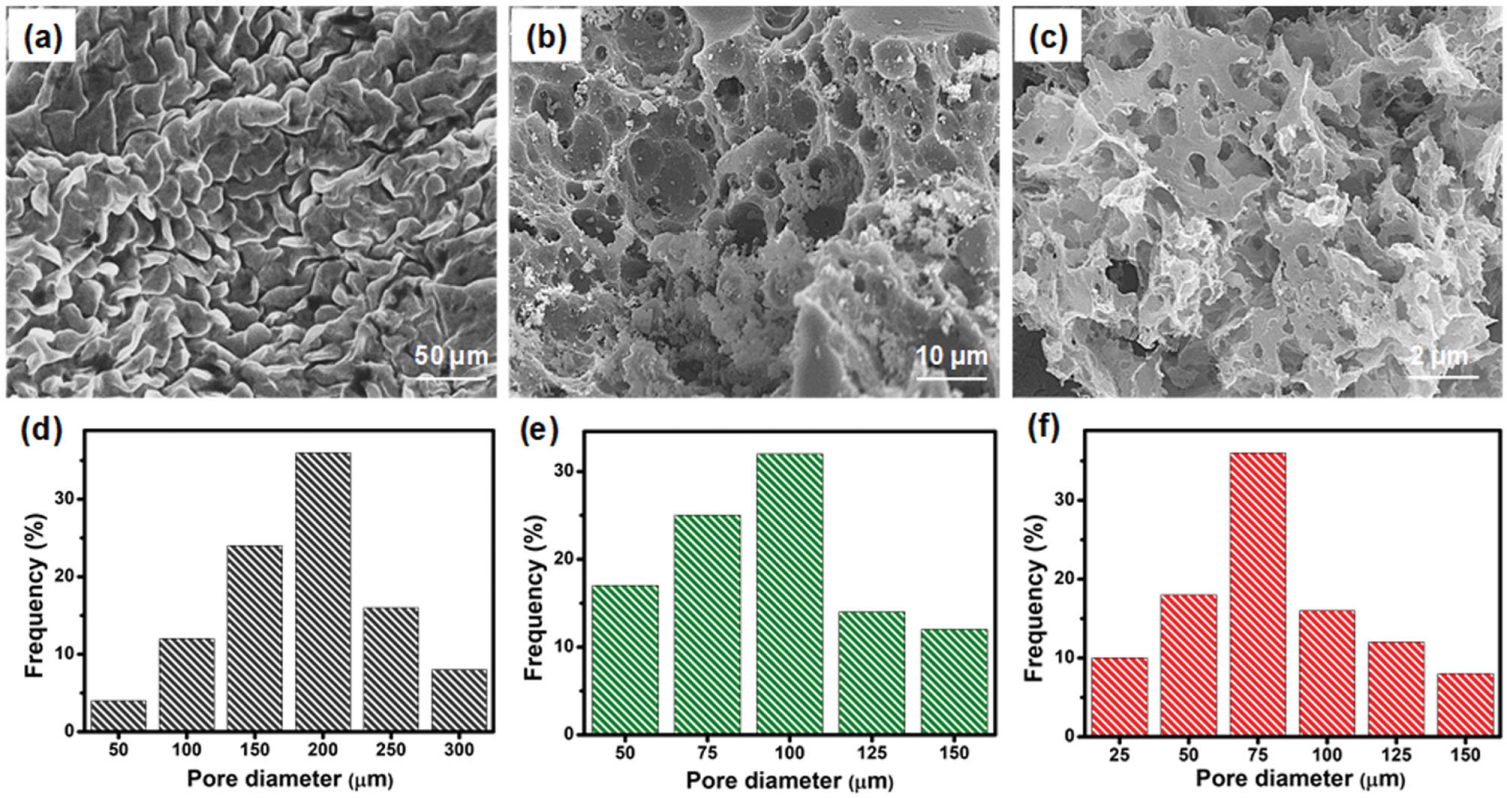
The scanning electron micrographs (a–c) and pore diameter (d–f) of the CS-GP hydrogels using various concentration of GP (0.5%, 1.0%, and 1.5%).

The morphology of the prepared three-dimensional (3D) hydrogels was examined in the freeze-dried state by scanning electron microscopy (SEM). SEM images of CS-GP hydrogels using various concentration of GP (0.5%, 1.0%, and 1.5%) as shown in Figure 1(a–c). The distribution range of pore size was assessed using ImageJ software (Bethesda, USA). It can be noted that the hydrogels are highly porous, with pore diameter ranging between 25 and 300 μm in Figure 1(d–f). In comparison, CS-GP-1, CS-GP-2 and CS-GP-3, consisting of 0.5%, 1.0%, and 1.5% of GP, show a smaller pore size but non-uniform size distribution (Leal-Egaña et al., 2011). The addition of various concentrations of GP noticeably affects the porous structure (Vo et al., 2020). Physically crosslinked CS produces open networks with the smallest pore dimensions (∼75 μm), whereas chemical crosslinking with GP produces open networks with the biggest pore dimensions (∼200 μm). The addition of GP to these compositions diminishes the pore diameters of the hydrogels and compact the microstructure (Kirchmajer et al., 2013). This can be explained by the fact that the hydrophobic contact is more extensive than chemical crosslinking. The larger pores in the CS-GP hydrogel will aid in cell development and proliferation, while the smaller pores will aid in the transfer of essential nutrients within the hydrogel (Muzzarelli et al., 2015). Cell migration will be aided by the pores that exist deep within the 3D network.

**Figure 2.**
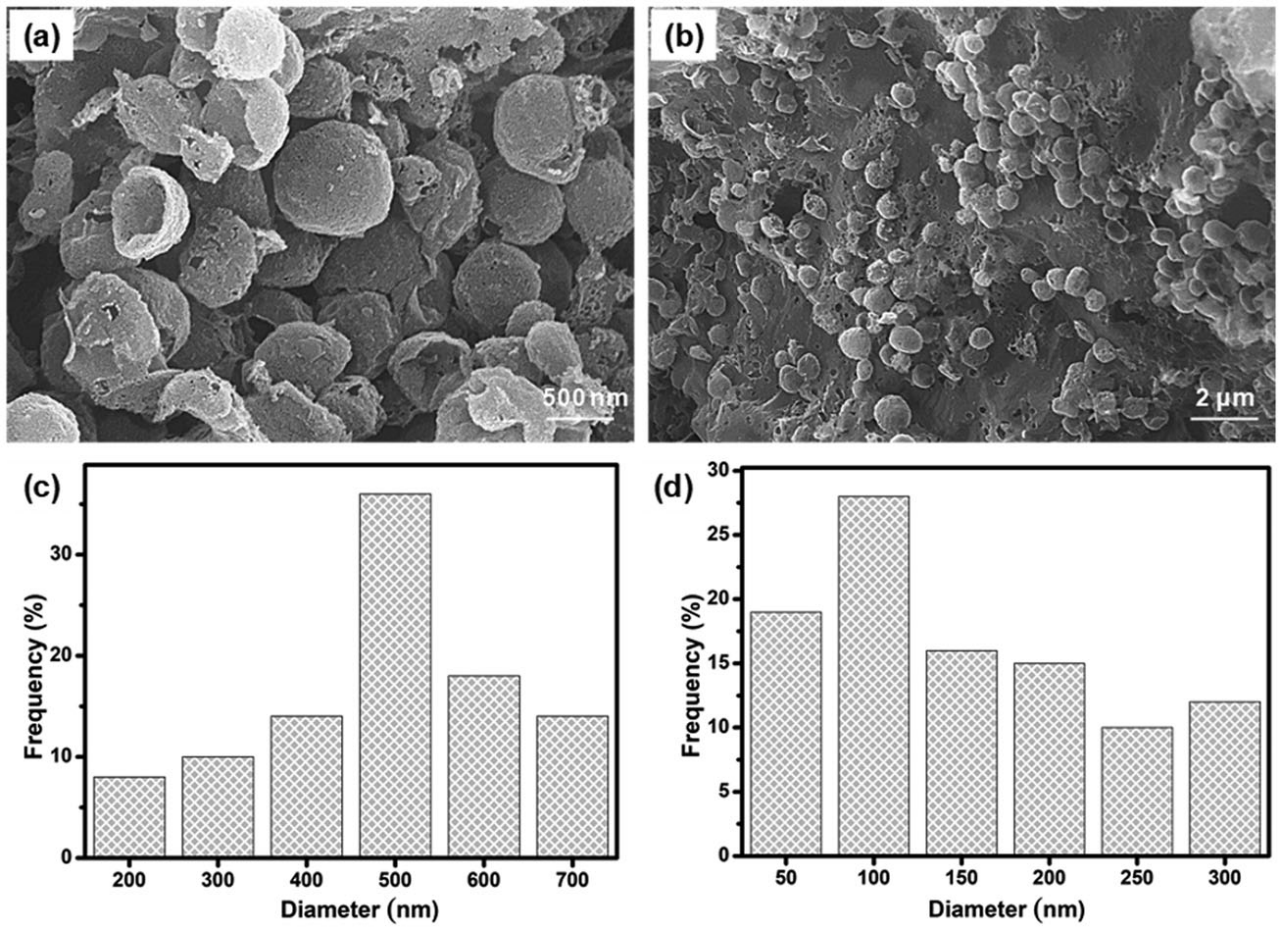
SEM micrographs of (a) PC nanocapsules (b) and CS-GP/PC polymeric hydrogel. The histogram of (c) PC nanocapsules (d) and CS-GP/PC polymeric hydrogel diameter.

As shown in Figure 2, PC nanocapsules were embedded within the CS-GP hydrogel matrix and the PC nanocapsules maintained their round morphology in the CS-GP hydrogel (Lee et al., 2017). The CS-GP/PC polymeric hydrogel possessed a more compact surface and smaller capsules size than that of PC nanocapsules, and the PC nanocapsules particles dispersed on the surface of CS-GP hydrogel pores homogenously (Crecente-Campo et al., 2019). Furthermore, regular distribution of the capsules and the variation in their size can be also demonstrated. These images revealed a 3D structure of CS-GP/PC polymeric hydrogel with different capsules sizes in the range of 50–700 nm. The average size of PC nanocapsules and CS-GP/PC polymeric hydrogel was 500 nm and 100 nm, respectively (Deng et al., 2020). Adding the PC nanocapsules particles into the CS-GP hydrogel crosslinking networks leads to denser and finer structures. Therefore, the appropriate size at 100 nm in the PC nanocapsules blend CS-GP hydrogel is more favorable for cellular seeding, proliferation, and formation of extracellular matrix. These results consist of the characterizations of FT-IR and XRD analysis.

### 3.2. Swelling ratio of hydrogels

**Figure 3.**
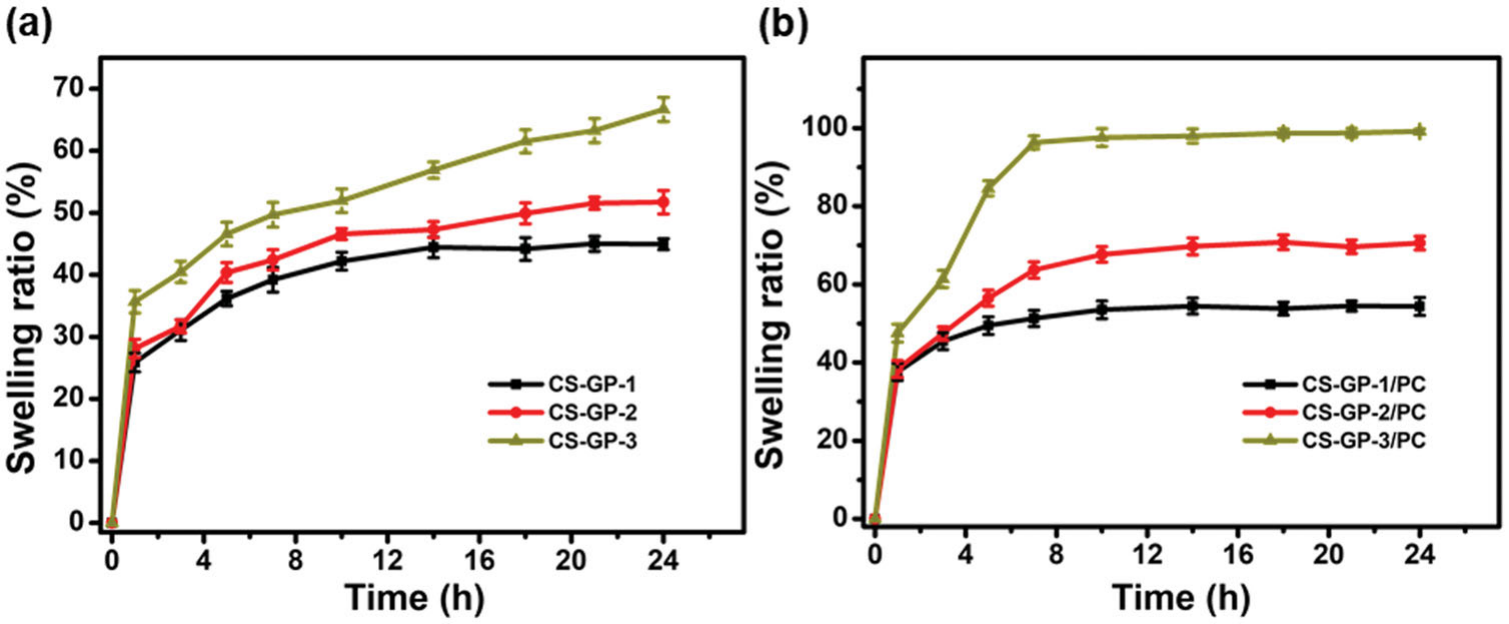
Swelling ratio of the (a) CS-GP hydrogels using various concentration of GP (0.5%, 1.0%, and 1.5%) and (b) CS-GP hydrogels using various concentration of GP (0.5%, 1.0%, and 1.5%) in presence of PC nanocapsules at room temperature.

The swelling ability of the hydrogel is a significant consideration when evaluating its properties as composites for tissue-engineered and precise drug delivery systems (Carbinatto et al., 2014). The swelling ratio of CS-GP hydrogel and CS-GP/PC polymeric hydrogel are shown in Figure 3. When the adding concentration of GP at 0.5%, 1.0%, and 1.5%, the swelling ratio of CS-GP-1, CS-GP-2, and CS-GP-3 become 44.9 ± 0.90%, 51.7 ± 1.90% and 66.6 ± 1.95%, respectively. Because of the presence of polarizable groups such as –OH, –NH_2_, and –COOH, the hydrogel is a hydrophilic polymer with a high degree of swelling (Liu et al., 2021). Furthermore, the hydrophilicity of these formulations is improved by the PC nanocapsules granules distributed in CS-GP hydrogel and between pores (Bodenberger et al., 2016).

In comparing the swelling ratios of CS-GP-1/PC, CS-GP-2/PC, and CS-GP-3/PC, we find that the swelling ratio of hydrogels increases properly as the concentration of GP increases from 0.5% to 1.5%. The CS-GP hydrogel presented a fast initial swelling rate, but the CS-GP/PC polymeric hydrogel displayed a greater swelling rate than the CS-GP hydrogels (Zhang et al., 2020). Before 5 h, the swelling rate determined for the CS-GP/PC polymeric hydrogel was 84.5 ± 1.90%, demonstrating a polymeric ability that can be understood by the high hydrophilic nature of PC nanocapsules within CS-GP hydrogels. The swelling ability of all the produced hydrogels enhanced as the GP content and PC nanocapsules were increased. By increasing the surface hydrophilic nature of the hydrogels, PC nanocapsules can aid in the diffusion of water molecules (Mantha et al., 2019). This may be due to the hydrophilic PC nanocapsules’ contribution to the CS-GP hydrogels. As a result, this research offers a potential method for producing a CS-GP/PC polymeric hydrogel with strong mechanical characteristics and water content.

### 3.3. *In vitro* degradation

**Figure 4.**
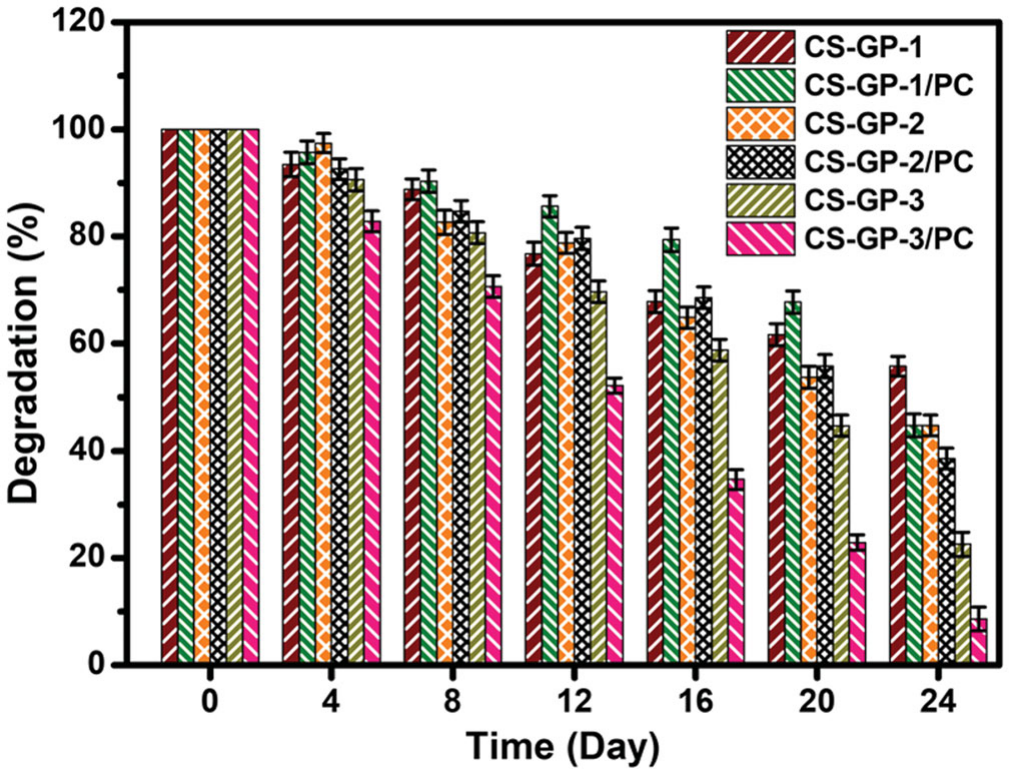
*In vitro* degradation profile of CS-GP hydrogels using various concentration of GP (0.5%, 1.0%, and 1.5%) and in presence of PC nanocapsules incubated in PBS (pH 7.4) at 37 °C.

*In vitro* degradation profile of CS-GP hydrogels using various concentration of GP (0.5%, 1.0%, and 1.5%) and in presence of PC nanocapsules incubated in PBS (pH 7.4) at 37 °C. Figure 4 depicts the weight decrease for the various samples. During the entire degradation time, the weight loss of the CS-GP hydrogels and CS-GP/PC polymeric hydrogel is found to be slow (Lewandowska-Łańcucka et al., 2019). The remaining amount of CS-GP hydrogels and CS-GP/PC polymeric hydrogel was 22.5 ± 2.25% and 8.6 ± 2.21%, respectively, at the end of the 28-day testing period, as shown in Figure 4. The rates of deterioration of CS-GP hydrogels containing 1.5% GP are much higher than those of CS-GP hydrogels containing 0.5% or 1.0% GP. It is clear how the higher crosslinker density results in a slower degradation rate. Furthermore, a larger cross-linking agent amount can improve the scaffold’s cross-link density, limiting enzyme absorption into particular polymer chain locations and eventual breakdown. With the inclusion of PC nanocapsules, the degradation rate of the CS-GP/PC polymeric hydrogel was increased (Frank et al., 2014). The findings confirmed the formulation’s biodegradability, which is critical for long-term drug release.

### 3.4. Rheological properties

**Figure 5.**
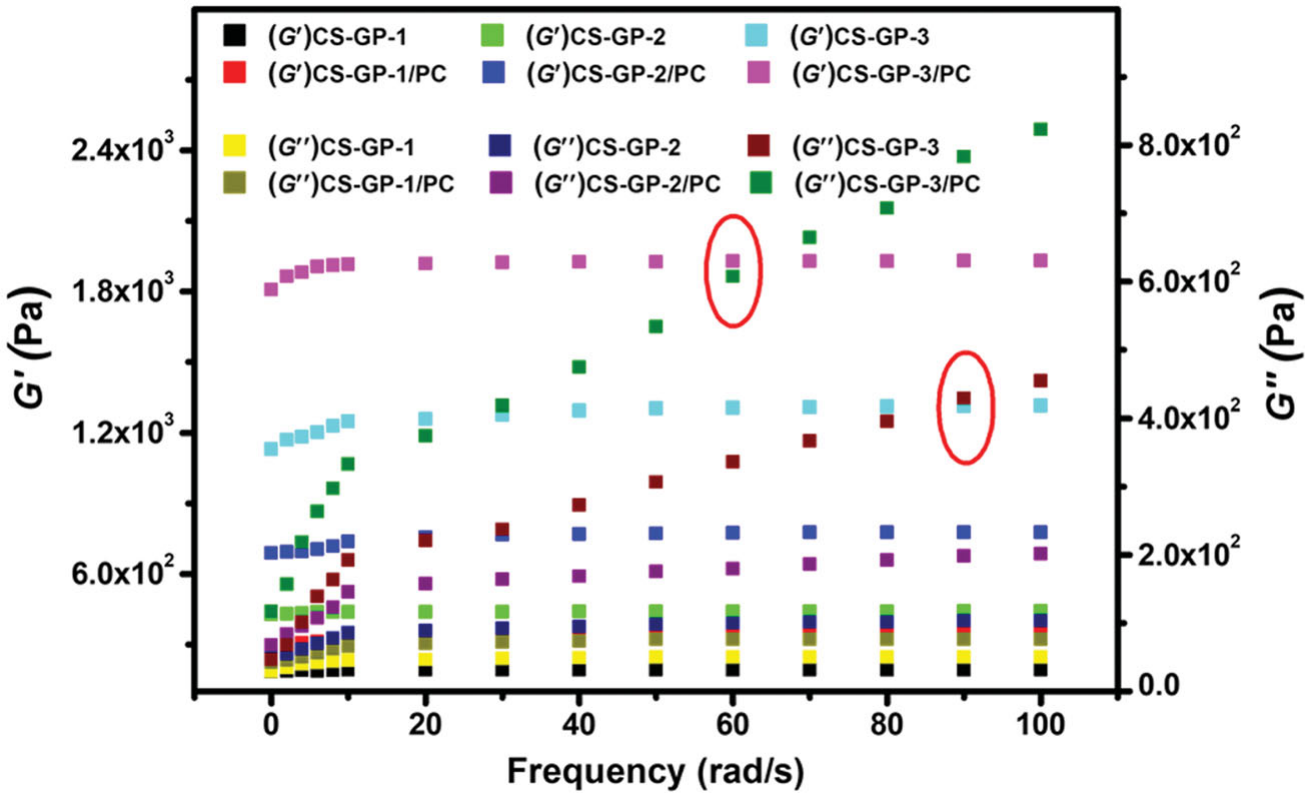
Frequency dependence of the storage modulus (G′) and loss modulus (G′′) versus frequency at 37 °C of CS-GP hydrogels using various concentrations of GP (0.5%, 1.0%, and 1.5%) in presence of PC nanocapsules.

The characteristics of CS-GP hydrogels and CS-GP/PC polymeric hydrogels are commonly characterized using rheological assessment. The storage modulus G′ and loss modulus G′′ are given as a function of frequency in a frequency sweep analysis, as shown in Figure 5. The frequency sweep of the fabricated CS-GP hydrogels employing varying concentrations of GP is shown in Figure 5 (Moura et al., 2007) (0.5%, 1.0%, and 1.5%). Over the whole angular frequency range (0.1–100 rad/s), the G′ and G′′ indicate a plateau zone, however, the G′ is only weakly reliant on frequency. G′ values are almost frequency-dependent across the testing range for all hydrogels and are substantially bigger than G′′ values at all frequencies, which is a hallmark trait of a ‘strong’ hydrogel (Kokol et al., 2021).

Furthermore, the G′ values for CS-GP/PC polymeric hydrogels are significantly greater than those for CS-GP hydrogels, indicating that PC nanocapsules may adequately strengthen CS-GP hydrogels. This means that CS-GP hydrogels with PC nanocapsules act more like solids and that the elastic behavior of the CS-GP/PC polymeric hydrogel improves as the GP content increase. Furthermore, the G′ values in CS-GP hydrogels and CS-GP/PC polymeric hydrogels increase as the GP concentration increase, which could be due to the attachments of the PC nanocapsules with the CS-GP hydrogel systems. It is noticeable that the gel point of the two moduli (CS-GP-3 and CS-GP-3/PC) with higher frequency is moved to lower times/frequency. The measured loss factor curves coincide with a single point in round marked as shown in Figure 5. This point is frequency independent and represents the gel-point of the sample. The CS-GP-3 and CS-GP-3/PC gel point is shifted from about 90–60 rad/s. This indicates the presence of restructuring arrangement of the CS-GP/PC polymeric hydrogel when a strain is applied, but no expressive reorganization was found as time progressed. However, the G′ value of the CS-GP/PC was higher than the G′′ value.

### 3.5. FTIR analysis

**Figure 6.**
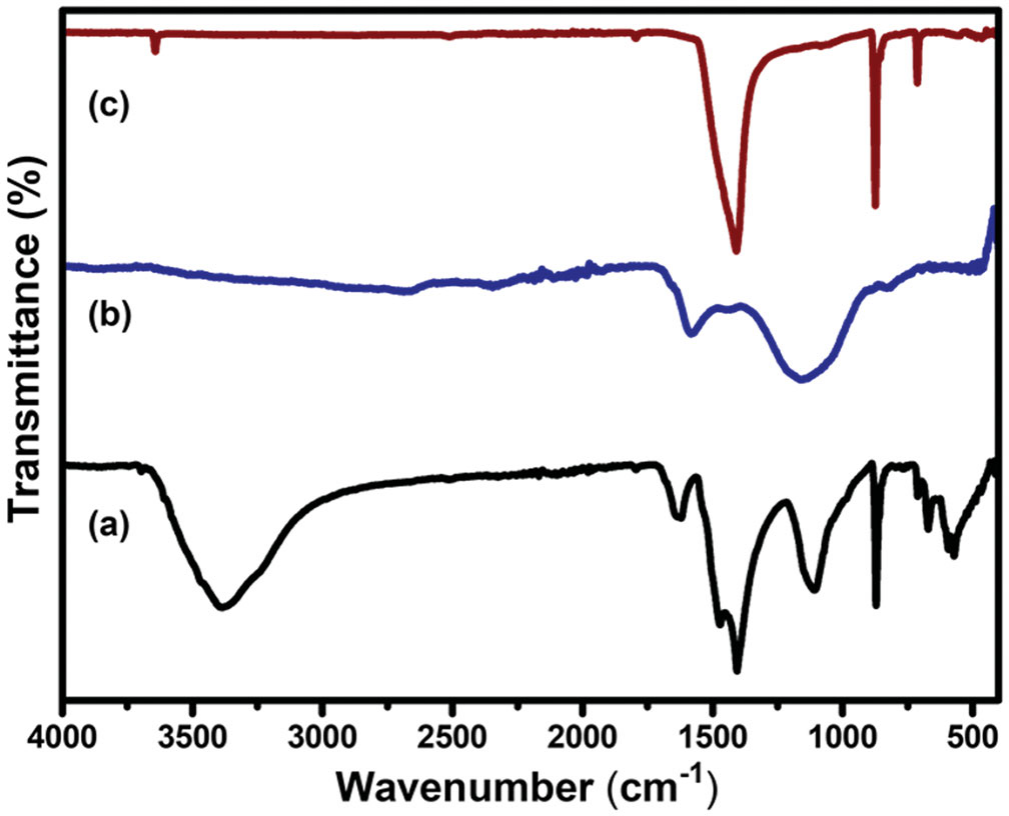
FTIR spectra of (a) CS-GP hydrogels, (b) PC nanocapsules, and (c) CS-GP/PC polymeric hydrogel.

FTIR spectra of CS-GP hydrogels, PC nanocapsules, and CS-GP/PC polymeric hydrogel are presented in Figure 6. The nucleophilic interaction of the amino group of CS on the olefinic carbon atom at C-3 of GP coupled to a glucosamine unit occurs during the polymerization of GP with CS (Racine et al., 2017). The amide II band at 1546 cm^−1^, which is indicative of N-H deformation, is likely attributable to the creation of secondary amides as a result of the reaction between the GP ester and hydrogen bonding and the CS amino groups after copolymerizing with GP (Dimida et al., 2017). C=O stretch in secondary amides was blamed for the peak at 1633 cm^−1^. Furthermore, the CS and CS-GP hydrogels displayed the characteristic C=O peaks at 1650 and 1580 cm^−1^, with the blue shift ascribed to the formation of amide linkages (Riaz et al., 2018). Moreover, absorptions from C–N stretching vibrations and C–OH stretching vibrations, which are more common after crosslinking with GP, can be attributed to the increase in peaks at roughly 1400 and 1000 cm^−1^. This result showed that GP and CS cross-linked to synthesize the CS-GP hydrogel. The absorption frequencies of the hydroxyl groups, C–H bonds, and ester groups emerge at 3295, 2921, and 1736 cm^−1^ in the FT-IR spectra of PC nanocapsules, respectively (Diyanat et al., 2019). The spectra of CS-GP/PC polymeric hydrogels showed considerable alterations in contrast to the frequencies of CS-GP hydrogels. Because the mechanisms engaged in the crosslinking reaction in the presence of the enzyme are the same involved in the crosslinking of PC nanocapsules. The disappearance of the band correlating to the primary amino groups after crosslinking was found in the FTIR spectrum of the CS-GP/PC polymeric hydrogel, which is consistent with the results evident for covalent CS-GP hydrogels containing PC nanocapsules (Xu et al., 2015). Changes in peaks were observed in the crosslinked PC nanocapsules loaded CS-GP hydrogels compared with pristine CS-GP hydrogels indicated successful crosslinking between CS and GP using PC nanocapsules (Garnica-Palafox & Sánchez-Arévalo, 2016).

### 3.6. XRD analysis

**Figure 7.**
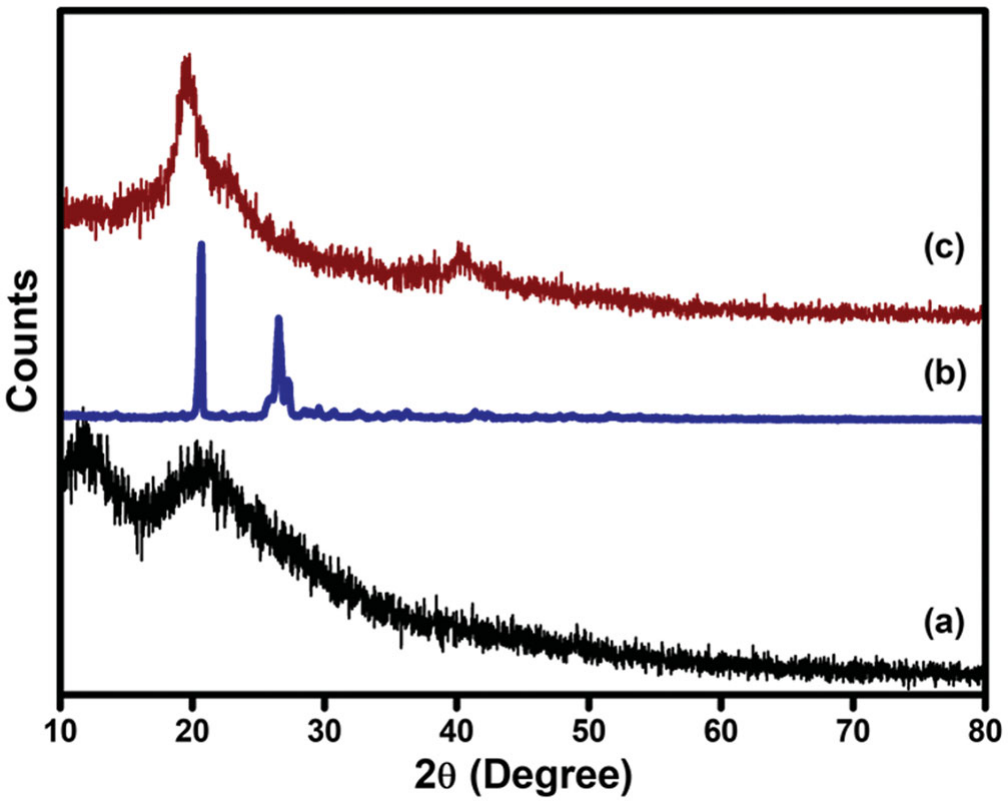
XRD pattern of (a) CS-GP hydrogels, (b) PC nanocapsules, and (c) CS-GP/PC polymeric hydrogel.

XRD patterns of CS-GP hydrogels, PC nanocapsules, and CS-GP/PC polymeric hydrogels are presented in Figure 7. The diffractogram of CS-GP hydrogels obtained broad peaks at 2*θ* of 11.8° and 23.1° (Ubaid & Murtaza, 2018). As can be seen, the chemical crosslinking with GP leads the resources to lose crystalline nature. A diffractogram of amorphous material was seen in all of the CS-GP hydrogels examined. On the other hand, the PC nanocapsules X-ray pattern showed strong peeks with high intensity at 2*θ* = 21.3° and 26.8°, indicating high crystallinity nature (Tavares et al., 2017). In addition, PC nanocapsules loaded CS-GP hydrogels showed broad peaks at 2*θ* = 21.9° and a new peak appeared at 2*θ* = 21.9°, representing the amorphous nature of CS-GP/PC polymeric hydrogel. Furthermore, as compared to those with crosslinked CS-GP/PC polymeric hydrogel, PC nanocapsules had higher and sharper crystalline peaks, indicating that the crystalline structure of PC nanocapsules had changed following crosslinking.

### 3.7. EE and DL of CS-GP/PC polymeric hydrogel

It is well documented that CS-GP hydrogel and CS-GP/PC polymeric hydrogel have excellent encapsulation efficiencies (EE) and drug loading (DL) mainly due to their porous structures and numerous functional groups (Tian et al., 2021). The EE and DL values of the fabricated CS-GP hydrogel and CS-GP/PC polymeric hydrogel were calculated, and the results obtained were summarized in Table 1. EE and DL of the CS-GP hydrogel and CS-GP/PC polymeric hydrogel range from 69.8 ± 3.65% to 86.8 ± 2.15% and 2.9 ± 0.40% to 4.5 ± 0.41% respectively, exposed the significantly enhanced in the BPV loading efficiency from CS-GP hydrogel to CS-GP/PC polymeric hydrogel (Tronci et al., 2014). The higher amount of hydrophilic groups in the composites assisted to improve intramolecular H-bonding, which helped to encapsulate the drug (Yin et al., 2009). The attained outcomes presented that, the BPV drug is loaded in the pores of the CS-GP/PC polymeric hydrogel and increasing % of EE and % DL with increasing the content of BPV drug may be ascribed to the presence of a large number of pores in the CS-GP/PC polymeric hydrogel.

**Table 1.** Encapsulation efficiency and drug loading of BPV added to CS-GP hydrogel and CS-GP/PC polymeric hydrogel.

Sample	EE (%)	DL (%)
CS-GP/BPV	69.8 ± 3.65	2.9 ± 0.40
CS-GP/PC/BPV	86.8 ± 2.15	4.5 ± 0.41

### 3.8. *In vitro* drug release from the hydrogels

**Figure 8.**
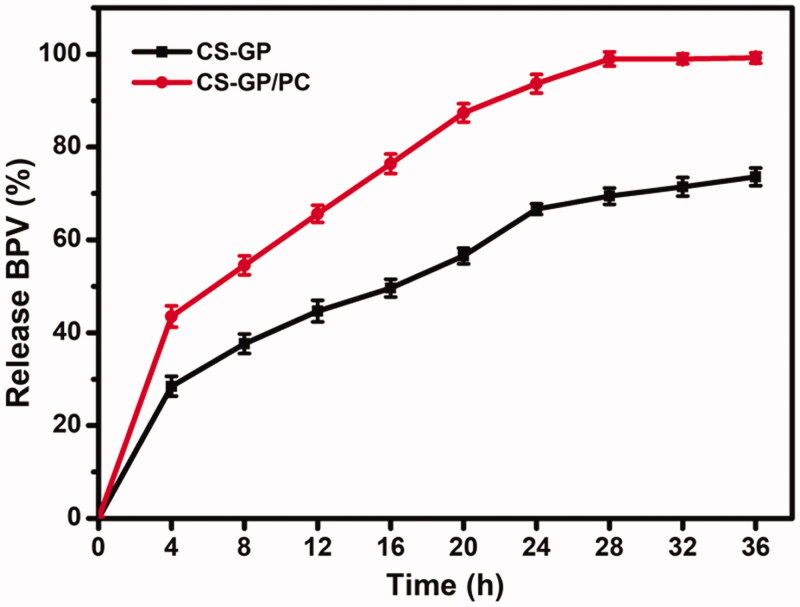
*In vitro* BPV Drug release profile (%) CS-GP hydrogel and CS-GP/PC polymeric hydrogel under sink conditions at pH 7.4.

We developed the study by considering the BPV effects on the release behavior of the PC nanocapsules-loaded CS-GP hydrogel. The aqueous release media which exhibited adequate sink conditions (required solubility and stability) for BPV were selected to study accelerated *in vitro* drug release. As shown in Figure 8, cumulative drug release of CS-GP hydrogel and CS-GP/PC polymeric hydrogel was investigated in PBS at 37 °C for 36 h (Gull et al., 2019). During the 16th hour, the cumulative release of BPV from CS-GP hydrogel and CS-GP/PC polymeric hydrogel was continuous and steady, reaching 49.6 ± 1.90% and 76.3 ± 2.10%, respectively. Similarly, after 16 h, drug release became more consistent and slower, with ultimate release rates of 73.6 ± 1.90% and 99.2 ± 1.12%, respectively. We believe that the BPV drug spread easily from the aqueous network structures, resulting in the burst release in the beginning (Hu et al., 2021). When the release was 99%, however, the systems levels practically reached equilibrium after 12 h. These results are interesting because, as seen in the drug release studies carried out in the sink condition at pH 7.4, the CS-GP hydrogel and CS-GP/PC polymeric hydrogel expands and/or breaks down, which could promote a much faster release of the drug. Because of the dual-stage drug loading strategy or the slow degradation rate of the hydrogel, this controlled release of BPV drug has the effect of reducing drug distribution to healthy tissues while enhancing drug accumulation at the tumor site for a longer time (Grillo et al., 2010). We hypothesize that this double-barrier construction will prevent BPV medication loss during early burst release, reducing hazardous side effects. It is worth noting that the goal of this research was to develop a system that could release the medicine quickly after administration, assuring rapid anesthesia and boosting patient compliance.

### 3.9. *In vitro* cytotoxicity assay

**Figure 9.**
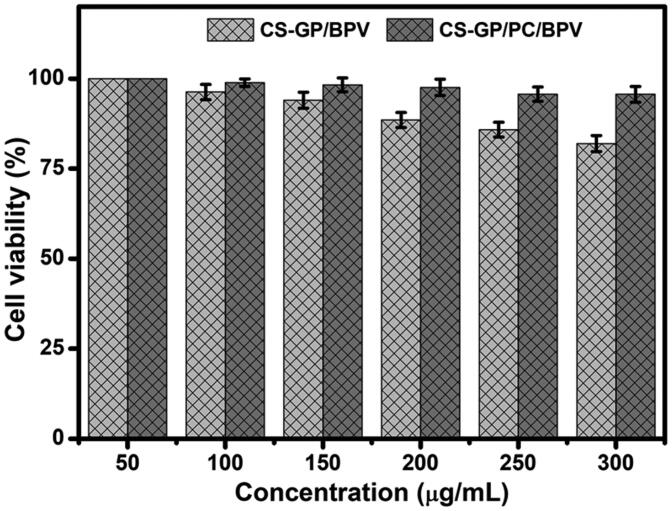
*In vitro* cytotoxicity of CS-GP hydrogel and CS-GP/PC polymeric hydrogel using different concentrations (50–300 μg/mL) against 3T3 cells was confirmed by a CCK-8 assay.

The CCK-8 assay was performed in human fibroblast 3T3 cells for 24 h to validate the biocompatibility and nontoxicity of CS-GP hydrogel and CS-GP/PC polymeric hydrogel, as well as the anesthetic effects of BPV in the dosage form (Li et al., 2021). Although several researchers have demonstrated that GP is a safer cross-linker than glutaraldehyde, the compatibility of composite hydrogel formulations must be validated (Liang et al., 2003). After 24 h of treatment, the cell survival of drug doses showed a dose-dependent response in the concentration ranges of 50 to 300 µg/mL, the dose-dependent cytotoxicity of cells treated with BPV by the CS-GP hydrogel and CS-GP/PC polymeric hydrogel was examined, as shown in Figure 9.

The maximal cell viability was recorded at 200 µg/mL (97.5 ± 2.25%), and the viability marginally reduced when the concentration was increased to 300 µg/mL (95.6 ± 2.20%). As a result, the produced hydrogel has good biocompatibility and might be used as a drug delivery device that is nontoxic. The findings suggest that by inducing apoptotic cell death, BPV could enhance the therapeutic efficacy of anesthetic effects drugs (Xuan et al., 2016). The BPV drugs were delivered into the cells by the CS-GP/PC polymeric hydrogel and showed anesthetic pain reduction efficiency, which could explain why the polymeric hydrogel has a better analgesic activity impact that involves exposure to the cells. *In vitro* cytotoxicity data show that the clinical effects of BPV in the CS-GP hydrogel composition are much more significantly increased, that is, it has greater cytotoxicity than the CS-GP/PC polymeric hydrogel composition.

### 3.10. *In vitro* skin permeation

**Figure 10.**
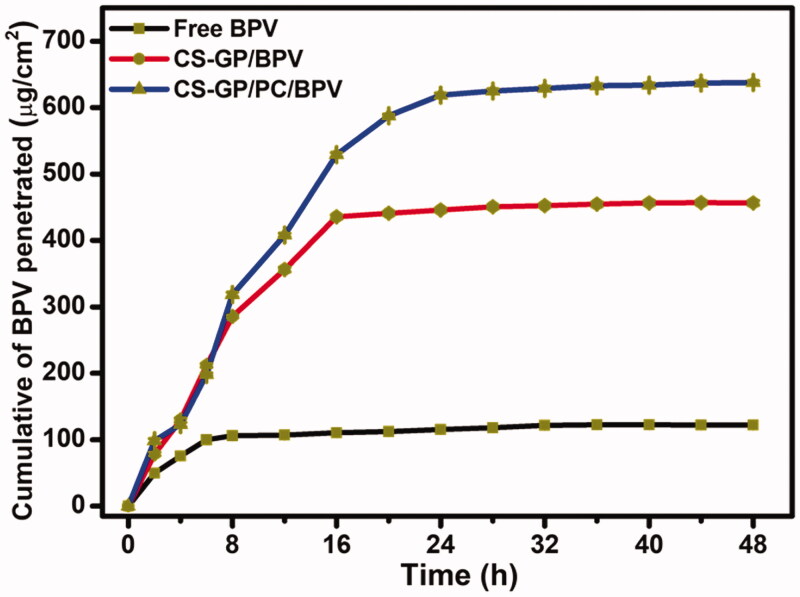
*In vitro* permeation studies of free BPV, CS-GP/BPV, and CS-GP/PC/BPV solution on the skin of mice.

The ability of free BPV medicines and BPV-loaded CS-GP/PC polymeric hydrogel to penetrate the skin of mice was demonstrated *in vitro* utilizing skin permeation measurements. It is often assumed that the diffusion of a molecule in the skin can be represented as the inverse square of its molecular weight (Yue et al., 2018). The BPV permeation results with different formulations were shown in Figure 10. Figure 10 shows that the permeation of BPV in CS-GP/PC polymeric hydrogel was much more sufficient than BPV in CS-GP hydrogel solutions (Zhang, Xu, et al., 2018). The amount of BPV absorbed through the skin from the CS-GP hydrogel and CS-GP/PC polymeric hydrogel was much higher than that of free BPV in this investigation. CS-GP hydrogel and CS-GP/PC polymeric hydrogel enhanced BPV permeability 3-fold and 5-fold in 24 h, respectively, when compared to BPV solution (Yang et al., 2019). The findings showed that CS-GP/PC polymeric hydrogel formulas have a higher skin permeation ability, which could be due to their similarity to skin lipids, the high specific surface area for drug absorption due to their smaller size in the nanometric range, the presence of a solid matrix, and biocompatibility, which makes them useful for long-term skin administration (Qi et al., 2021). We created PC nanocapsules in this investigation and used them to make the CS-GP/PC polymeric hydrogel. The hydrophilic patch component of PC nanocapsules allows them to hydrate and diffuse through skin cells, whereas the hydrophobic patch region improves their permeability across the skin (Pignatello et al., 2009; Wang, Wang, et al., 2016; Wang, Zhang, et al., 2016; Zhang et al., 2016). This is consistent with the findings that modifying PC nanocapsules enhanced BPV skin permeation effectiveness.

### 3.11. *In vivo* anesthetic pain relief

**Figure 11.**
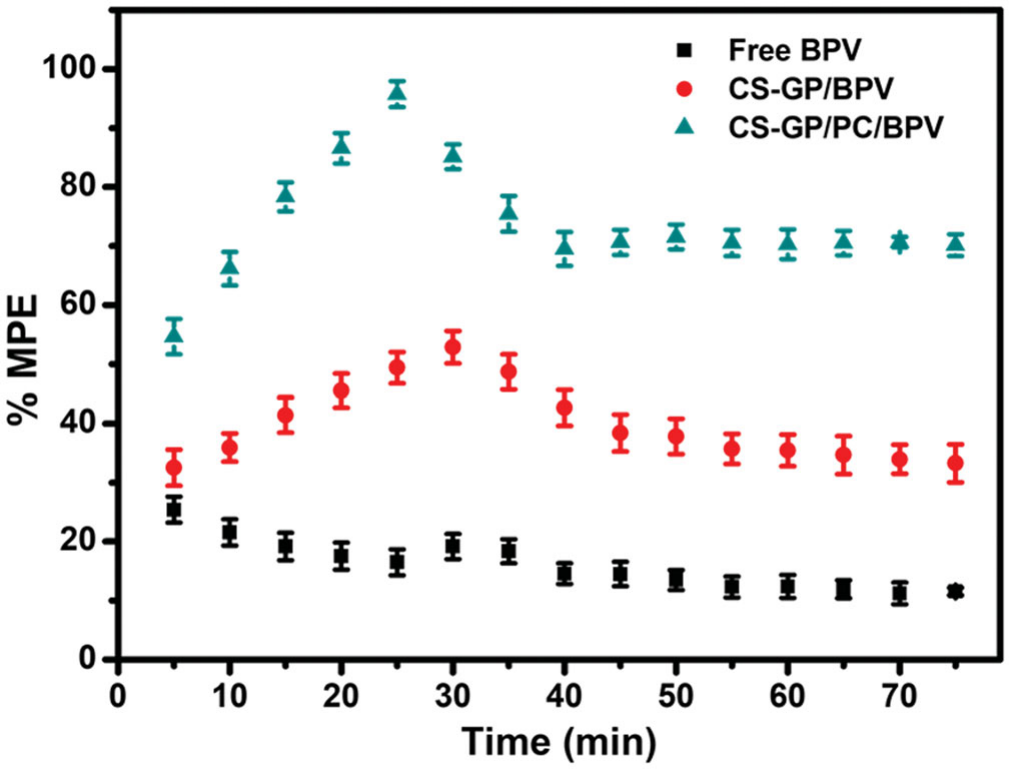
*In vivo* tail-flick test for the evaluation of the local anesthetic effects of free BPV, BPV-containing CS-GP, and BPV-containing CS-GP/PC formulations.

Free BPV showed a quick but short-term pain reduction impact *in vivo* anesthetic and pain relief trials, with the effect steadily decreasing throughout the test (Zhang et al., 2017). The Tail-flick test was used to assess the effectiveness of anesthetic pain alleviation *in vivo* (Figure 11). During the test, a blank CS-GP hydrogel and a CS-GP/PC polymeric hydrogel without BPV had no effect. Free BPV had a quick but short-term pain-relieving impact, which gradually faded throughout the test. When compared to BPV loaded CS-GP hydrogel and free BPV, all of the BPV loaded CS-GP/PC polymeric hydrogels exhibited more persistent and pronounced anesthetic effects (Melo et al., 2018; da Silva et al., 2021). We also demonstrated that the anesthetic effects of the BPV-loaded CS-GP/PC polymeric hydrogel in mice lasted longer and were more persistent, with over 95.7 ± 2.20% recovery recorded at time intervals of 20–30 min. Additionally, they may cause harm to the animal skin, making it much more difficult to assess the anesthesia effects. BPV incorporation in CS-GP/PC polymeric hydrogel resulted in a prolonged anesthetic effect, which could be explained by verifying the previous hypothesis that CS-GP/PC polymeric hydrogel causes the two anesthetics to accumulate in the upper skin layers, avoiding unnecessary flux and creating a reservoir capable of extending skin retention time. CS-GP/PC/BPV had the highest percentage of maximum possible effect (%MPE) and the longest-lasting anesthetic pain relief *in vivo* among the numerous BPV formulations studied, therefore the composition of CS-GP/PC/BPV is established for the maximum anesthetic pain relief performance (Muniz et al., 2018). This phenomenon could be observed that a higher BPV level aids *in vivo* anesthetic and pain reduction (Mihalache et al., 2020). These results indicated that adding a small dose of BPV to a large composition could boost the anesthetic efficacy of BPV. The goal of employing hydrogel compositions is to reduce drug concentration and improve therapeutic efficiency. The final formulation showed good anesthetic and pain reduction efficiency *in vivo* and might be used as a promising anesthesia system, according to the findings.

### 3.12. Local tissue response

**Figure 12.**
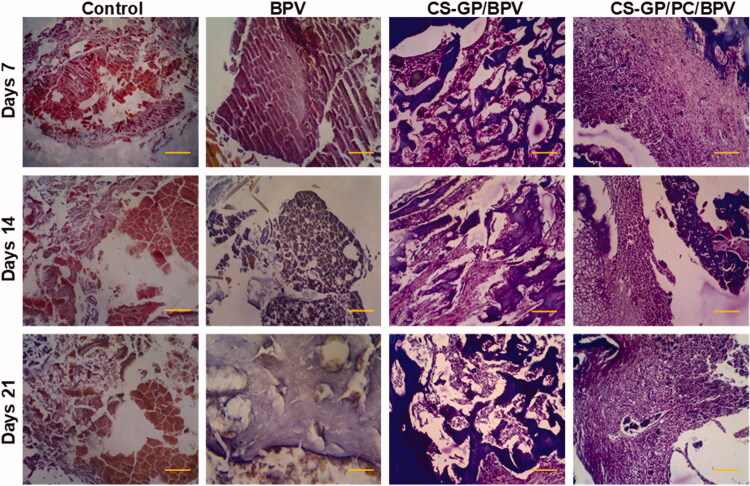
Histopathological evaluation of the anti-inflammatory effects of BPV-loaded CS-GP hydrogel and CS-GP/PC polymeric hydrogel on 7, 14, and 21 days were stained with hematoxylin and eosin (H&E) (scale bar = 100 μm).

**Figure 13.**
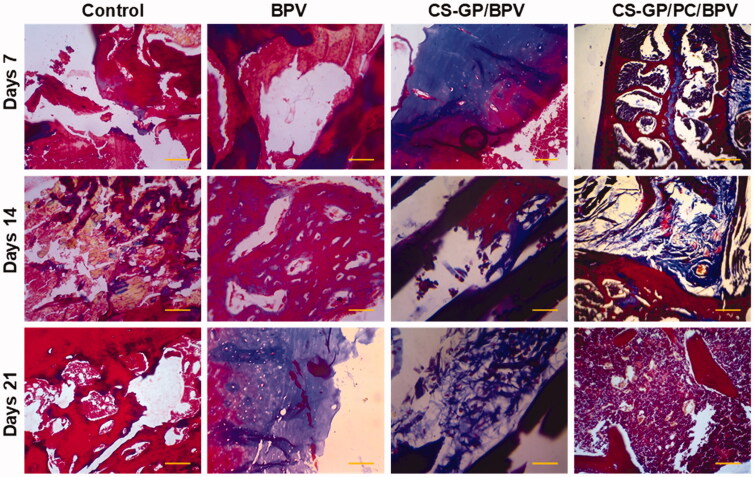
Histopathological evaluation of the anti-inflammatory effects of BPV-loaded CS-GP hydrogel and CS-GP/PC polymeric hydrogel on 7, 14, and 21 days were stained with Masson’s trichrome stain (MTS) (scale bar = 100 μm).

All animals surrounding tissue were removed 7, 14, and 21 days following the original operations. There was no obvious tissue damage in any of the test groups. Figures 12 and 13 show representative microscopic pictures of Hematoxylin and eosin (H&E) and Masson trichrome (MST) stained tissue sections. These tests were performed on the tissues of each group, with consistent results in all tissue samples. The animals in the non-treated groups (healthy animals) do not have any irritation on their skin. The controlled release compositions-treated CS-GP/BPV and CS-GP/PC/BPV groups had mid-level inflammation, but the CS-GP and CS-GP/PC groups exhibited only mild inflammation (Zhang, Ning, et al., 2018), as illustrated in Figures 12 and 13.

The burst or persistent release of the BPV medication produced from the disintegration of the CS-GP/PC polymeric hydrogel may have induced the tissue reactions prompted by the controlled release compositions (Jiang et al., 2020). The majority of the inflammatory cells and infection were seen in the soft tissue surrounding the residues, with very little inflammation in the muscle. In addition, the CS-GP/PC/BPV hydrogels animal groups reveal that any other tissue damages are greatly reduced (Foley et al., 2013; Khanal et al., 2018). Furthermore, negligible inflammation was found 7, 14, and 21 days after injection, demonstrating that the inflammatory response generated by the BPV drug-containing CS-GP/PC polymeric hydrogel compositions is reversible.

## 4. Conclusions

Pain management with local anesthetics is usually limited by the short duration of analgesia. BPV was entrapped in CS-GP/PC polymeric hydrogel, which was constructed using CS-GP/PC/BPV as hydrophilic lipid shell and PC nanocapsules as the hydrophobic polymeric core. In this study, we developed a CS-GP/PC polymeric hydrogel made from CS cross-linked GP-loaded PC nanocapsules. CS-GP/PC polymeric hydrogel is denser than CS-GP hydrogel, thus allowing for a high swelling ratio, slower degradation, and superior rheological properties. The CS-GP hydrogel has a homogenous and porous structure, with elliptical pores of non-uniform diameters, as indicated by SEM. PC nanocapsules in CS-GP/PC polymeric hydrogel had a small size, narrow distribution, and excellent cross-linking hydrogels surface. The resulting CS-GP/PC polymeric hydrogel did not have harmful effects in 3T3 fibroblast cells, and *in vivo* testing demonstrated that the anesthetic effect of the BPV-loaded CS-GP/PC polymeric hydrogel was implemented after 24 min with no further side effects. The long-lasting *in vivo* anesthetic effect in rats was consistent with the prolonged BPV drug release characteristic of the CS-GP/PC polymeric hydrogel. The CS-GP/PC polymeric hydrogel had a higher ex vivo penetration rate than the CS-GP hydrogel and BPV drug solution. Finally, a BPV-loaded CS-GP/PC polymeric hydrogel successfully obtained quick-onset, long-lasting infiltration anesthesia in rats without causing significant toxicity, indicating a potential route to long-lasting local anesthetics development.

## Data Availability

The authors confirm that the data supporting the findings of this study are available within the article. Raw data that support the findings of this study are available from the corresponding author, upon reasonable request.
